# The impact of healthcare visit timing on reported pertussis cough duration: Selection bias and disease pattern from reported cases in Michigan, USA, 2000–2010

**DOI:** 10.1186/s12879-016-1852-0

**Published:** 2016-09-29

**Authors:** Jennifer K. Knapp, Mark L. Wilson, Susan Murray, Matthew L. Boulton

**Affiliations:** 1 Department of Epidemiology, School of Public Health, University of Michigan, Ann Arbor, 48109 Michigan USA; 2 Department of Biostatistics, School of Public Health, University of Michigan, Ann Arbor, 48109 Michigan USA; 3 Department of Internal Medicine, Division of Infectious Diseases, University of Michigan Medical School, Ann Arbor, 48109 Michigan USA

**Keywords:** Pertussis, Cough, Antibiotics, Epidemiology, Selection bias

## Abstract

**Background:**

Pertussis is a potentially serious respiratory illness characterized by cough of exceptionally long duration of up to approximately100 days. While macrolide antibiotics are an effective treatment, there is an ongoing debate whether they also shorten the length of cough symptoms. We investigated whether public health surveillance data for pertussis, in which cases are identified at diagnosis, are potentially affected by selection bias and the possible consequences for reported cough duration.

**Methods:**

Data on 4,794 pertussis cases reported during 2000–2010 were extracted from the Michigan Disease Surveillance System, a statewide, web-based communicable disease reporting system, to specifically investigate increased duration of cough observed in pertussis patients with delayed initial healthcare visit. A simulated population of cases was derived from the observed surveillance data and truncated week-by-week to evaluate the effects of bias associated with stratification on timing of antibiotics.

**Results:**

Cases presenting for medical evaluation later in the clinical course were more likely to have experienced delayed antibiotic therapy and longer average cough duration. A comparable magnitude of increasing cough duration was also observed in the simulated data. By stratifying on initial medical visit, selection bias effects based on timing of healthcare visit were demonstrated.

**Conclusions:**

Stratifying or controlling for the timing of the initial case identification and accompanying antibiotic treatment can create artificial patterns of observed cough duration. In surveillance data, differences in symptom duration may arise from selection bias and should not be presumed to be related to early antibiotic treatment.

## Background

Pertussis, caused by the bacterium, *Bordetella pertussis,* and clinically characterized by a uniquely prolonged cough, represents a potentially serious respiratory illness that has experienced recent increases in incidence levels not observed in the U.S. since the 1950′s [1]. The U.S. Centers for Disease Control and Prevention (CDC) conducts ongoing monitoring of pertussis cases through state-based public health surveillance, and has utilized a pertussis case definition requiring a minimum 14-day cough, plus the presence of at least one of the following cough attributes: post-inspiratory whoop, coughing paroxysms, or post-tussive vomiting [2]. The pathology of cough symptoms is not well understood, but appears to be the result of toxin-mediated virulence factors of *B. pertussis* infection [3].

Treatment of pertussis with macrolide antibiotics reduces the risk of transmission by effectively eliminating the pathogen from patients in less than 3 days [4, 5]. This decreased risk of transmission is the primary reason for antibiotic therapy, both in cases and in exposed individuals. However, whether or not the timely use of antibiotics shortens cough duration and the clinical course of disease continues to be debated. While the CDC pertussis treatment bulletin maintains that antibiotic use results in a shorter course of disease [6], a number of studies that include case follow-up until cessation of coughing show no effect of antibiotic therapy on cough duration, even when antibiotics (like the macrolide, azithromycin) are prescribed prophylactically, that is before the onset of cough [4, 7–10].

Public health surveillance for pertussis typically identifies cases at the point that an individual seeks medical attention for a cough, which is the dominant clinical feature. However, healthcare seeking behavior for pertussis is undoubtedly affected by both the severity and duration of symptoms, including cough. This complicates analyses, because stratifying on a variable within the causal pathway between the exposure and outcome can induce a care-seeking bias. For example, if adult patients wait twice as long as children to seek medical care for an illness and the diagnostic sensitivity is associated with length of illness, then children will be over-represented in the surveillance when compared to the population of ill persons. Controlling for (or stratifying on) the timing of the medical visit makes it appear that age has an effect on diagnostic sensitivity, when this may be due entirely to differences in care-seeking behavior. Since passive surveillance data identifies cases who choose to go for medical care, the identified cases may not represent all infections in the population at large, and care-seeking behavior creates an important source of selection bias.

Stratifying pertussis cases based on the timing of the initial medical visit may also create an unintentional case-exclusion bias, as cases who delay the initial visit are under-represented in the data. Indeed, they may be less likely to seek care at all and, therefore, remain undetectable through clinical-or laboratory-based surveillance. This exclusion of milder infections from the surveillance database becomes more pronounced among cases that present at later stages; especially around the time the illness begins to resolve. As a result of this form of selection bias, cases who delay the initial medical evaluation appear to have a more prolonged clinical presentation. Studies using public health surveillance data rarely consider the potential impact of care-seeking selection bias or case-exclusion bias and, thereby, can contribute to a mistaken impression that earlier treatment shortens cough duration [11–13]. This purported effect is magnified when accompanied by incomplete reporting of the outcome, such as cough duration in pertussis cases.

**Fig. 1:**
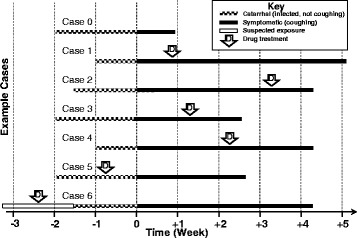
Sample pertussis case clinical histories, to illustrate data limitations, biases and truncation. These six cases are illustrative of the general timeline of symptom development and clinical care. Time zero is the day of cough onset. Some cases seek medical attention because of a potential exposure (Cases 5 and 6). While both receive antibiotics prior to cough onset, but only case 6 received truly prophylactic treatment. However, this analysis cannot reliably distinguish between these cases in the prophylactic group of observed cases. The cough lengths of such estimates are included in the observed estimates. However, the theoretical analysis cannot distinguish any prophylactic cases from those who received medical treatment in Week + 1 (Case 1). The analysis of this paper created a dataset that replicated the distribution of cough duration (*black* lines) based on the mean and standard deviation of the natural log transformed surveillance data. The date of drug treatment is our proxy for first medical visit, and is the potential source of bias we are testing as cases are both identified and subsequently stratified on this time point. By calculating the mean of the theoretical distribution we estimated the average cough for anyone who could visit the doctor in the first week of cough. By excluding cases, we can determine who is still eligible to have their first drug treatment in Week + 2. Therefore we must exclude all individuals who already sought care (Cases 1, 5 and 6); this is care-seeking bias. Additionally individuals who have already stopped coughing would also be excluded; this is case exclusion bias (Case 0). This process of excluding cases with events to the left of the cut-point is call left-truncation. By calculating the mean cough length of everyone remaining in the theoretical population after truncation, we can estimate the new mean duration of those who are eligible to see the physician in Week + 2

In this analysis, we compared the week-to-week differences in mean cough duration in the observed surveillance data. Cases present in a variety of temporal sequences and thereby enter into surveillance reporting in ways that may contain bias. The epidemiological patterns of cases were considered with regard to when they started symptomatic coughing, and when a health-care visit and drug therapy was begun (Fig. 1). In addition to summarizing the observed data, we used a novel simulation approach to create a theoretical data set and compared how stratification by timing of first physician visit (considered to be first antibiotic prescription) would affect the theoretical mean of cough duration. This creates a simulated population of ill-persons who are still coughing, and therefore eligible for a first medical visit, and theoretically are detectable by the surveillance system. We then compared the week-to-week differences of the surveillance data and the theoretical results to determine whether the incremental increase in cough duration of surveillance data could be explained by selection bias.

## Methods

### Data source

The study included cases of pertussis reported to the Michigan Department of Health and Human Services (MDHHS) during 2000 through 2010 as part of the state’s routine surveillance for reportable communicable diseases. All probable or confirmed pertussis case records were included if they met two criteria based on the dates provided in the records: 1) a cough duration could be calculated, and 2) duration of the cough at the time of the first medical appointment could be established. Probable cases of pertussis were those that met standard clinical criteria, whereas confirmed cases also included a positive laboratory test or were a close contact of another laboratory-confirmed case. The first reported date of antibiotic prescription was used as a proxy for the initial healthcare visit. These data were compared with a simulated data set that was generated from characteristics of the observed cases.

### Data simulation

The observed MDHHS data distribution was used to simulate a theoretical data set using the RAND function of SAS, which is a random-number function from a pseudo-random number generator. We specified a simulated sample of 5,000 cases, with a normal distribution of cough duration, based on the mean and standard deviation (SD) of natural-log transformed values of cough duration that were actually reported. The original cough duration had a log-normal distribution. The simulated data represent a theoretical population of ill persons. The simulation population was left-truncated in week-long increments, to exclude those whose cough resolved prior to week of first healthcare visit; this mimics the effects of delaying healthcare. The dataset was successively truncated (weeks were removed) at days 7, 14, 21 and 28, following cough onset. The retained data represents the week-by-week distribution of individuals remaining in the general ill population (illustrative examples in Fig. 1). Following each of the four truncations, the mean cough duration of the remainder of the population was re-calculated. By removing everyone in the simulated population whose cough resolves before day seven, for example, only those who are still ill and eligible to visit a physician in week 2 (days 7–13) remain (Fig. 1). The simulated population analysis can neither describe the effects of seeking medical care prior to coughing (prophylactic treatment) nor abstaining from antibiotics.

### Data analysis

The associations between the timing of the initial healthcare visit relative to cough onset and other disease characteristics in the MDHHS data were tabulated. We then compared the week-to-week differences within the theoretical data to the observed results, to determine whether the incremental increase in cough duration of surveillance data could be explained by selection bias.

A comparison is also made to determine whether there were significant differences between cases in week one against later weeks, week 0 data was excluded. Categorical comparisons were made using a chi-squared test, while comparisons of means were made using the Satterthwaite *T*-test. All data management was conducted using SAS version 9.2 (Cary, North Carolina, USA). The data acquisition and analysis plan for this study was approved by the Institutional Review Board (IRB) of the MDHHS and subsequently deemed exempt from review as secondary data analysis by the IRB of the University of Michigan.

## Results

There were 4,794 unique pertussis cases reported in Michigan during 1 January, 2000 through 31 December, 2010, of which 3,365 (70.1 %) had information recorded for both total cough duration and for duration of cough at initial clinical evaluation (or initial medical appointment). Reported cough duration of cases averaged 32.1 days (standard deviation (SD) = 23.7). Of the reported cases, 82.2 % were still coughing when the surveillance report was finalized. Virtually all pertussis cases (99.1 %) reported taking some type of antibiotic, the most common of which were Clarithromycin/Azithromycin (70.3 %), Erythromycin (14.6 %), and Amoxicillin (6.5 %).

Cases presenting later in the clinical course of disease for initial medical evaluation were experienced by definition delayed antibiotic therapy and also were more likely to have a) longer average cough duration (difference = 9.8 days 95 % CI (7.9–11.6), Satterwaithe *p*-value < .01), b) more accompanying cough attributes (difference = 5.4 %, *χ*^2^ = 21.8, *p <* .01), and c) treatment specifically with macrolide antibiotics (difference = 6.9 %, *χ*^2^ = 21.9, *p <* .01) (Table 1). These associations were also observed when stratifying by age (data not shown). However, cases who were prescribed antibiotics prior to cough onset (Table 1, Week 0), reported a mean cough duration of 28.7 days, although ~20 % of these cases were still coughing when the cough length was reported. This is cough duration was similar to that observed among those whose initial visit was in the first and second week of cough onset (Table 1).

**Table 1:** Pertussis cough characteristic trends associated with initial medical visit (week of antibiotic prescription) and simulated findings

Week of antibiotic prescription		Observed Percentage of records			Observed days		Simulated Days^a^	
	N^b^	Coughing at final interview % (95 % CI)	Macrolide antibiotic % (95 % CI)	Cough attribute % (95 % CI) ^c^	Cough duration mean (95 % CI)	Mean difference^d^	Cough duration mean (95 % CI)	Mean difference^d^
0^e^	65	80.0 (70.0–90.0)	65.6 (52.7–76.6)	81.5 (71.8–81.2)	28.7 (24.8–32.5)	N/A	N/A	N/A
1st	809	79.7 (76.9–82.5)	79.7 (76.0–81.7)	86.8 (84.4–89.1)	24.8 (23.4–26.1)	−3.9	26.1 (25.6–26.5)	N/A
2nd	1043	84.1 (81.9–86.3)	84.8 (81.9–86.3)	92.0 (90.3–93.6)	25.6 (24.6–26.5)	0.8	26.9 (26.4–27.3)	0.8
3rd	613	86.5 (83.7–89.2)	85.7 (82.0–87.7)	92.0 (89.9–94.2)	30.5 (29.2–31.9)	4.9	31.8 (31.3–32.3)	4.9
4th	341	83.9 (79.9–87.8)	89.6 (85.2–92.0)	92.7 (89.9–95.4)	34.8 (33.5–36.1)	4.3	38.8 (68.5–39.4)	7.0
5th^f^	494	76.1 (72.3–80.0)	89.2 (85.9–91.5)	92.7 (90.4–95.0)	58.3 (55.1–61.5)	23.5	46.1 (45.4–46.8)	7.3

*CI* confidence interval, *N* number

^a^ Simulated cough duration (number of days) was calculated using a hypothetical population distribution and applying the observed Michigan pertussis case data from actual surveillance, then removing people (left-truncating) 1 week at a time

^b^ N refers only to the observed data. Some characteristics were not reported in all surveillance records, therefore N varies

^c^ Cough attributes include cases having one or more of the following (proportion positive within the dataset): paroxysms (85.1 %), post-tussive vomiting (51.2 %), or whooping (37.0 %)

^d^ The difference of the mean cough duration of cases with their first medical visit in the current week (same row) compared to the previous week (previous row)

^e^ Week 0 cases had their first medical visit prior to the onset of cough, and received antibiotics prophylactically

^f^ The 5th week also contains all first medical visits in the fifth and following weeks

The mean of the individual cough duration values was 26.6 days (95 % Confidence Interval (CI) 26.0–27.1). Left-truncation of the simulated data showed that care-seeking bias results in longer mean cough duration with each successive week (Table 1). An average increase of 1 day of cough occurred between weeks 1 and 2, of 5 days between weeks 2 and 3, and of 7 days per week thereafter. This week-to-week analysis characterized the effects of truncation, or delayed care seeking, on a theoretical population independent of treatment, so that increasing cough length was not a result of antibiotic therapy. Instead, it would appear to be due to care-seeking bias. This care-seeking bias effect, demonstrated via truncation, parallels the difference in cough duration observed in the actual MDHHS data over the first 5 weeks, when more than three-quarters of cases sought medical care.

## Discussion

The dramatic increase in pertussis cases nationally presents an opportunity to develop more accurate characterization of cases through well-designed public health surveillance to better inform our understanding of risk and the role of treatment on case detection and the course of clinical disease [1]. Our study reveals that stratifying surveillance detected pertussis cases by timing of the initial medical visit creates the false impression of a trend of increasing cough length, independent of antibiotic therapeutic effects. If antibiotics could effect a shorter cough duration, then cases who sought medical attention earliest in their course of illness should have the shortest duration of cough. These would be those cases who used antibiotics prophylactically, before a cough began (Fig. 1, Cases 5 & 6). This was not evident in our analysis, and also other data [13]. Care-seeking bias would neither affect those cases who sought care prior to cough onset [8], nor those who did not use antibiotics [13], where the entire cough duration was reported. The pertussis cases who received antibiotics prophylactically (*N =* 65) were not of sufficient number to support additional analysis and, therefore, a simulation was conducted.

Our study aimed to evaluate whether care-seeking and case-exclusion biases in the pertussis surveillance data could explain the increased cough duration observed when the cases are stratified on the date of antibiotics, which is also the initial medical visit, and the point of inclusion in the dataset. Our examination of these selection biases demonstrated that truncation of a simulated ill population, like stratification of surveillance data according to the timing of antibiotics, produced similar week-to-week increases in mean cough duration. This suggests that the bias can be producing the longer mean cough observed in successively later weeks of the surveillance data.

Because the bias effects can account for the results of stratification, the increasing cough lengths of those who delay medical care is unlikely to be related to the specific effects of antibiotic treatment, which is supported in the literature [4, 7–10]. Perhaps cough duration is not dependent on the length of exposure to the bacterium and its toxins, but rather the extent of cellular damage and the slow speed of cellular regeneration (ciliated epithelial cells have a half-life of around 6 months) [14].

While the observed cases had a mean cough duration of 26.6 days, a study limitation was that 82 % of cases were reported as still coughing during the final surveillance case report. Some studies of surveillance data found a similar mean cough duration of around a month [15, 16], but others found a median to persist up to 7 weeks [11, 13]. If the median duration is 7 weeks, then it follows that the cases in our data set were followed for less than 7 weeks, as only 20 % reported complete cessation of cough. This length of follow-up should be sufficient for pertussis treatment and outbreak investigation. While having complete cough duration data would increase the mean duration of cough, it would not affect the premise of the study, which examines the effects of selection-bias or left-truncation on the mean cough duration of a theoretical population.

## Conclusions

Pertussis cases reported at the time of diagnosis, as is typical for public health surveillance, are subject to both care-seeking and case-exclusion biases. Our analysis demonstrates that the truncating simulation can account for the incremental differences in cough duration observed when pertussis cases are stratified according to week of first medical visit (when antibiotics are prescribed), therefore a selection bias exists. This bias can largely explain the increase in cough duration observed in surveillance data among patients who delay care, and is more parsimonious than an explanation based on the debated effects of antibiotic treatment.

## Abbreviations

CDC: Centers for disease control and prevention
CI: Confidence interval
IRB: Institutional review board
MDHHS: Michigan department of health and human services
SD: Standard deviation
